# *Psoralea corylifolia* L. Seed Extract Attenuates Methylglyoxal-Induced Insulin Resistance by Inhibition of Advanced Glycation End Product Formation

**DOI:** 10.1155/2019/4310319

**Published:** 2019-12-26

**Authors:** Cao-Sang Truong, Eunhui Seo, Hee-Sook Jun

**Affiliations:** ^1^College of Pharmacy and Gachon Institute of Pharmaceutical Science, Gachon University, Incheon 21936, Republic of Korea; ^2^Lee Gil Ya Cancer and Diabetes Institute, Gachon University, Incheon 21999, Republic of Korea; ^3^Gachon Medical Research Institute, Gil Hospital, Incheon 21565, Republic of Korea

## Abstract

Accumulation of advanced glycation end products (AGEs) in the body has been implicated in the pathogenesis of metabolic conditions, such as diabetes mellitus. Methylglyoxal (MGO), a major precursor of AGEs, has been reported to induce insulin resistance in both *in vitro* and *in vivo* studies. *Psoralea corylifolia* seeds (PCS) have been used as a traditional medicine for several diseases, but their potential application in treating insulin resistance has not yet been evaluated. This study is aimed at investigating whether PCS extract could attenuate insulin resistance induced by MGO. Male C57BL/6N mice (6 weeks old) were administered 1% MGO in their drinking water for 18 weeks, and the PCS extract (200 or 500 mg/kg) was orally administered daily from the first day of the MGO administration. We observed that both 200 and 500 mg/kg PCS extract treatment significantly improved glucose tolerance and insulin sensitivity and markedly restored p-Akt and p-IRS1/2 expression in the livers of the MGO-administered mice. Additionally, the PCS extract significantly increased the phosphorylation of Akt and IRS-1/2 and glucose uptake in MGO-treated HepG2 cells. Further studies showed that the PCS extract inhibited MGO-induced AGE formation in the HepG2 cells and in the sera of MGO-administered mice. PCS extract also increased the expression of glyoxalase 1 (GLO1) in the liver tissue of MGO-administered mice. The PCS extract significantly decreased the phosphorylation of ERK, p38, and NF-*κ*B and suppressed the mRNA expression of proinflammatory molecules including TNF-*α* and IL-1*β* and iNOS in MGO-administered mice. Additionally, we demonstrated that the PCS extract attenuated oxidative stress, as evidenced by the reduced ROS production in the MGO-treated cells and the enhanced expression of antioxidant enzymes in the liver of MGO-administered mice. Thus, PCS extract ameliorated the MGO-induced insulin resistance in HepG2 cells and in mice by reducing oxidative stress via the inhibition of AGE formation. These findings suggest the potential of PCS extract as a candidate for the prevention and treatment of insulin resistance.

## 1. Introduction

Insulin is an essential hormone produced by pancreatic *β*-cells, which acts to transport glucose from the bloodstream into cells for conversion into energy [1]. The reduction in the responsiveness of tissues to the normal action of insulin is referred to as insulin resistance [2]. Many studies have indicated that insulin resistance plays a key role in the development of type 2 diabetes mellitus. Insulin resistance is also strongly associated with several metabolic conditions, including diabetes, hypertension, and cardiovascular diseases [3–5]. Although the etiology of insulin resistance remains controversial, advanced glycation end products (AGEs) have been increasingly recognized as one of the contributing factors [6–9].

Methylglyoxal (MGO), a highly reactive dicarbonyl compound, is a major precursor of the AGEs. MGO mainly reacts with the arginine residues and, to a much lesser extent, with lysine residues, to form MGO-derived AGEs [10, 11]. Once AGEs are formed, these irreversible products interact with the receptors for AGEs (RAGE) resulting in inflammation and the elevated production of reactive oxygen species (ROS), which are also closely linked to insulin resistance [12]. AGEs contribute to two harmful factors, inflammation and oxidative stress, which lead to the development of insulin resistance. Thus, inhibition of AGE formation may be a potential strategy to counteract this condition.

*Psoralea corylifolia* L., commonly known as “Boh-Gol-Zhee” in Korea, has been widely used for the treatment of various pathological conditions, such as skin disorders, cancer, inflammatory diseases, neurodegenerative diseases, and kidney disease [13–15]. Every part of this plant is useful, with the seeds of *P. corylifolia* reported to contain six major components (bakuchiol, isopsoralen, psoralen, corylifolin, corylin, and psoralidin), all of which are potent antioxidants [15]. Particularly, bakuchiol protects against hepatic injury [16, 17]. Additionally, treatment with the *P. corylifolia* seed (PCS) extract can significantly improve hyperglycemia in streptozotocin-induced diabetes in C57BL/6 mice [18], and psoralen and isopsoralen have preventive effects against oxidative stress-induced beta-cell death and antitumor effects [18, 19]. However, the efficacy of the PCS extract in MGO-induced insulin resistance remains unexplored. Thus, this study is aimed at investigating whether the PCS extract attenuates MGO-induced insulin resistance *in vitro* and *in vivo* and determining the underlying mechanisms related to its effects.

## 2. Materials and Methods

### 2.1. Preparation of PCS Extract

PCS was purchased from an oriental drug store (Kwang Myung Dang Co., Ulsan, Korea), and the extraction was performed as described previously [18]. Briefly, the dried seeds (300 g) were ground into small pieces and extracted twice with 3 L of distilled water under reflux. The extract was stored in a freezer (-80°C) for 24 h before it was evaporated in vacuo to produce a dark brownish residue.

### 2.2. Animal Experiment

Male C57BL/6N mice (5 weeks old) were obtained from Orient Bio Inc. (Seongnam, Gyeonggi, Korea). All animals were subjected to a 12 h light/dark cycle and provided with food and water *ad libitum*. Mice were acclimatized for 1 week prior to experimentation and divided into the following five treatment groups based on the body weight and blood glucose levels: CON (control), MGO (1% in water, approximately 1460 mg/kg/day), MGO+PCS extract (200 mg/kg/day), MGO+PCS extract (500 mg/kg/day), and MGO+AG (30 mg/kg). Mice were treated with MGO in drinking water. PCS extract and AG were administered to the mice by oral gavage for 18 weeks. The body weight, food intake, and water intake were recorded daily, and the blood glucose level was measured every week. At the end of the experiment, half the mice in each group were stimulated with insulin (intraperitoneal injection, 1.5 U/kg) for 10 min before sacrifice (*n* = 8, each group).

### 2.3. Oral Glucose Tolerance Test (OGTT) and Insulin Tolerance Test (ITT)

OGTT and ITT were performed at week 18 of the experiment. In the OGTT, after a 16 h fasting period, mice were orally administered a glucose solution (2 g/kg). Blood glucose levels were measured using a glucometer after 30, 60, 90, and 120 min of glucose load. In the ITT, following a 4 h fast, mice were intraperitoneally injected with insulin solution (1.5 U/kg). Blood glucose level was recorded after 30, 60, 90, and 120 min of the insulin injection. OGTT was performed 3 days after the ITT.

### 2.4. Chemicals

Dulbecco's modified Eagle's medium- (DMEM-) high glucose and fetal bovine serum (FBS) were purchased from Welgene (Gyeongsangbuk-do, South Korea). Insulin human and MGO solution were obtained from Sigma-Aldrich (St. Louis, MO, USA). Humulin was purchased from Eli Lilly (Indianapolis, IN, USA). Bovine serum albumin, Fraction V (BSA), was purchased from MP Biomedicals (Irvine, CA, USA). Skim milk powder was obtained from BioShop Canada Inc. (Burlington, ON, Canada). Chemiluminescent horseradish peroxidase (HRP) substrate was purchased from Millipore (Billerica, MA, USA). Antibodies against *β*-actin (8457), insulin receptor substrate 1 (IRS1; 2390), p44/42 extracellular signal-regulated kinase mitogen-activated protein kinase (p44/42 ERK MAPK; 9102), p-Akt (4060), p-ERK (9101), p38 (9212), p-p38 (4511), and p-nuclear factor-kappa B (p-NF-*κ*B; p65) were purchased from Cell Signaling Technology (Beverly, MA, USA). Antibodies against phospho-IRS1/2 (Y^612^) (sc-17195-R), Akt1/2/3 (sc-81434), and GLO1 (sc-101537) were obtained from Santa Cruz Biotechnology (CA, USA). The anti-AGE (ab23722) antibody was obtained from Abcam (Cambridge, MA, USA). The human/mouse/rat serum albumin antibody (MAB1455) was purchased from R&D Systems (Minneapolis, MN, USA).

### 2.5. Cell Culture

HepG2 cells were cultured in DMEM containing 10% FBS and 100 units/mL penicillin and streptomycin. Cells were maintained under standard cultured conditions (37°C in a humidified 5% CO_2_ atmosphere).

### 2.6. Cell Viability Assay

The D-Plus™ CCK cell viability assay kit (Dongin LS, Seoul, South Korea) was used to measure HepG2 cell viability. Cells were seeded in 96-well plates (5 × 10^3^ cells/well) and incubated overnight for attachment. The cells were then treated with various concentrations of MGO or PCS extract for 24 h and 48 h. In a time-dependent test, the cells were treated with MGO (1 mM) for 0 h, 3 h, 6 h, 12 h, and 24 h. After treatment, the medium was changed, and CCK solution was added to the wells, followed by a 2 h incubation. The absorbance was measured at 450 nm using a Synergy™ 2 Multi-Mode Microplate Reader (BioTek Instruments, Winooski, VT, USA).

### 2.7. Glucose Uptake Assay

Glucose uptake was measured using the glucose uptake colorimetric assay kit (BioVision, Inc., Milpitas, CA, USA). HepG2 cells were seeded in 96-well plates at a density of 5 × 10^3^ cells/well and incubated overnight for attachment. The cells were starved using serum-free media for 12 h, followed by treatment with MGO (1 mM) and various concentrations of the PCS extract (50, 100, 200, and 500 *μ*g/mL) for 12 h. Cells were then starved for glucose using Krebs-Ringer-Phosphate-HEPES buffer containing 2% BSA for 40 min and stimulated with insulin (1 *μ*M) for 20 min. Next, 2-deoxyglucose (2-DG) was added, and the cells were incubated for 20 min. The optical density was measured at 412 nm using a microplate reader.

### 2.8. AGE Formation Assay

The assay was performed as described previously [20, 21]. Briefly, BSA (5 mg/mL) was mixed with 1 mM of MGO in phosphate-buffered saline (PBS, pH 7.4) containing 0.02% sodium azide in the absence or presence of PCS extracts at concentrations of 50, 100, 200, and 500 *μ*g/mL. Aminoguanidine (AG, 1 mM) was used as a positive control. The solutions were incubated at 37°C for 7 days. The experiment was conducted in duplicate to compare the results. The formation of AGEs was determined by measuring fluorescence of the solution in a 96-well black plate, at excitation/emission wavelengths of 355/460 nm using a multiplate reader at *t* = 0 and after 7 days of incubation.

### 2.9. Measurement of ROS Production

The level of ROS was measured using the CM-H_2_DCFDA dye (Invitrogen, Carlsbad, CA, USA). CM-H_2_DCFDA was dissolved in dimethylsulfoxide to obtain a 10 mM dye, which was then diluted in PBS containing Ca^2+^ and Mg^2+^ to achieve the final concentration of 10 *μ*M. Cells were seeded in a 96-well black plate at a density of 5 × 10^4^ cells/well. After overnight incubation, cells were pretreated with various concentrations of the PCS extract and subsequently treated with MGO (1 mM) for 1 h. The cells were then fixed in 10% neutral-buffered formalin for 10 min at room temperature, followed by staining with 10 *μ*M CM-H_2_DCFDA for 30 min at 37°C. After staining, cells were washed twice with PBS containing Ca^2+^ and Mg^2+^, and the plate was covered with aluminum foil. Fluorescent intensity was measured immediately at an excitation/emission wavelength of 495/527 nm using a fluorescence microplate reader.

### 2.10. Measurement of Glutathione Peroxidase (GPx) Activity

Liver tissue was homogenized with cold buffer (50 mM Tris-HCl, pH 7.5, 5 mM EDTA, and 1 mM dithiothreitol), and the antioxidant activity of GPx in the liver tissue was measured using the glutathione peroxidase assay kit (Cayman Chemical, Ann Arbor, MI, USA).

### 2.11. Quantitative Real-Time RT-PCR (qRT-PCR) Analysis

Total RNA was isolated from 8 mg of fresh liver tissue using RNAiso Plus (TaKaRa Bio Inc., Shiga, Japan), according to the manufacturer's instructions. The cDNA was synthesized using PrimeScript 1st strand cDNA synthesis kit (TaKaRa Bio Inc.) and was used as the template for the qRT-PCR. PCR was performed using the SYBR Premix Ex Taq II, ROX plus (TaKaRa Bio Inc.). Primer sequences are shown in Table 1. qRT-PCR was carried out for 10 min at 95°C, 40 cycles of 15 sec at 95°C, and 1 min at 60°C. qRT-PCR data was obtained as the threshold cycle (C_t_) value, and the gene expression was calculated by *ΔΔ*Ct.

### 2.12. Western Blotting

HepG2 cells were seeded into 6-well plates at a density of 1.6 × 10^5^ cells/well and incubated overnight before treating with MGO and PCS extract. The cells were then stimulated with insulin (0.5 *μ*M) for 30 min. The cells and liver tissue were homogenized using a motor-driven tissue grinder. Protein was extracted using a lysis buffer (M-PER™ mammalian protein extraction reagent) (Thermo Fisher Scientific, Waltham, MA, USA) containing a protease inhibitor cocktail and a phosphatase inhibitor cocktail (Sigma-Aldrich). For western blot analysis using a serum sample to detect AGEs, serum samples were diluted by 10x with PBS and mixed with 5x sample buffer. The protein was then separated by SDS-PAGE and transferred to a nitrocellulose membrane (GE Healthcare Life Sciences, Buckinghamshire, UK), which was then blocked with 5% nonfat skim milk or 5% BSA. The membrane was incubated with the specific primary antibodies, followed by washing three times with Tris-buffered saline containing Tween (TBS-T, 1%), and then incubated with HRP-conjugated secondary antibodies. The membrane was washed and then was detected and quantified with the ChemiDoc XRS+ system using Image Lab software (Bio-Rad, Hertfordshire, UK).

### 2.13. Statistical Analyses

Data are expressed as mean ± SD or SEM. One-way and two-way analyses of variance were performed using ANOVA analysis followed by Bonferroni's multiple comparisons test in GraphPad Prism software (GraphPad, La Jolla, CA, USA). Significance was determined at *p* values < 0.05.

## 3. Results

### 3.1. PCS Extract Improves Glucose Tolerance and Insulin Sensitivity in MGO-Administered Mice

**Figure 1:**
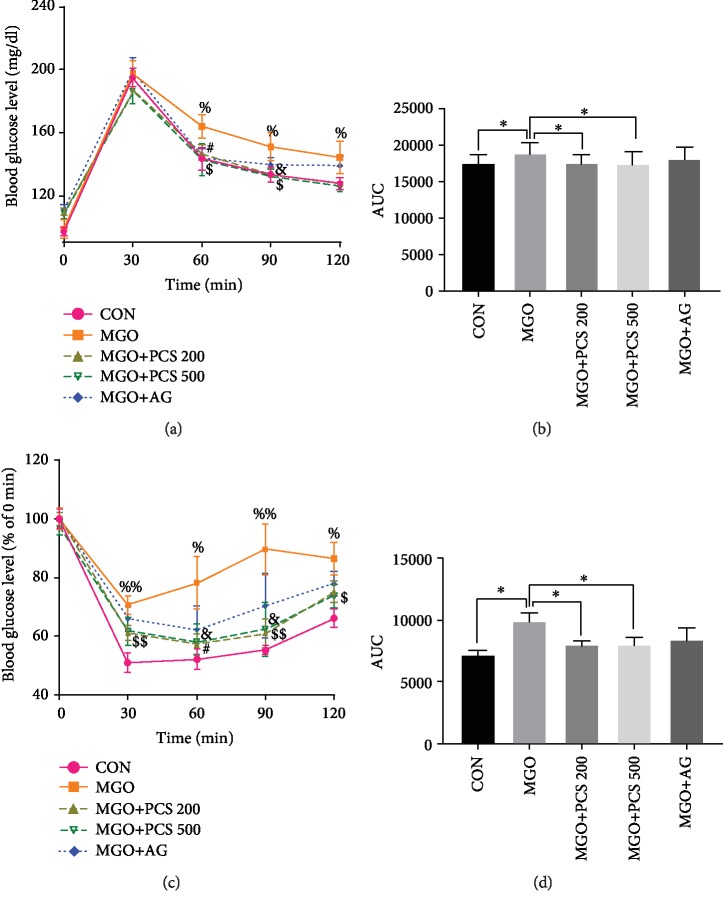
PCS extract improves glucose tolerance and insulin sensitivity in MGO-administered mice. Oral glucose tolerance test (OGTT) and insulin tolerance test (ITT) were performed at week 18 of the experiment. Blood glucose levels (a) and area under the curve (AUC) (b) of OGTT. Blood glucose levels (c) and AUC (d) of ITT. Data are shown as mean ± SEM (*n* = 8/group); ^∗^*p* < 0.05 in AUC (b, d). ^%^*p* < 0.05 CON vs. MGO. ^%%^*p* < 0.01 CON vs. MGO. ^&^*p* < 0.05 MGO vs. MGO+PCS 200.^$^*p* < 0.05, ^$$^*p* < 0.01 MGO vs. MGO+PCS 500. ^#^*p* < 0.05 MGO vs. MGO+AG. CON: control; MGO: methylglyoxal; MGO+PCS 200: methylglyoxal+PCS extract 200 mg/kg; MGO+PCS 500: methylglyoxal+PCS extract 500 mg/kg; MGO+AG: methylglyoxal+aminoguanidine.

To investigate whether the PCS extracts have beneficial effects on MGO-induced insulin resistance, mice were orally administered the PCS extract (200 or 500 mg/kg) for 18 weeks while simultaneously receiving 1% MGO in the drinking water. Blood glucose levels were significantly higher in MGO-administered mice at 60 min and 90 min after the glucose load (Figure 1(a)). In addition, calculation of the glucose area under the curve (AUC) revealed that AUC was significantly higher in the MGO-administered group as compared to the CON group, and treatment with the PCS extract at 200 and 500 mg/kg significantly reduced AUC after the glucose load (Figure 1(b)). Moreover, the administration of aminoguanidine (AG) significantly decreased the blood glucose level in MGO-administered mice (Figures 1(a) and 1(b)). In the ITT, insulin sensitivity was markedly reduced after 18 weeks of MGO treatment, as demonstrated by the higher blood glucose levels, compared to the CON group after insulin injection (at 30, 90, and 120 min). This reduced insulin sensitivity was attenuated by the administration of 200 mg/kg PCS extract, and significant results were observed with the 500 mg/kg dose of PCS. The AG treatment did not show significant hypoglycemic effects in the MGO-administered mice (Figures 1(c) and 1(d)).

### 3.2. PCS Extract Recovers Insulin-Stimulated p-Akt and p-IRS1/2 Expression in MGO-Administered Mice

**Figure 2:**
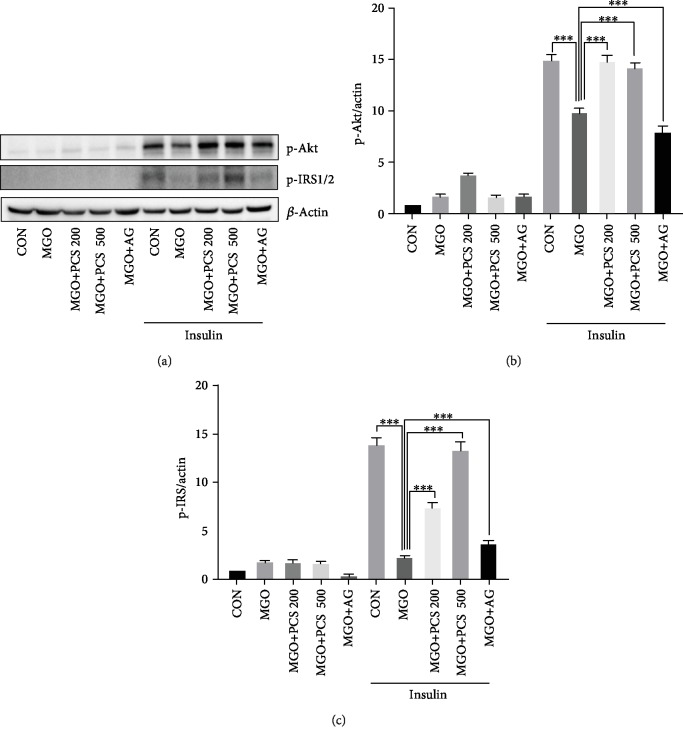
PCS extract treatment increases expression of insulin-stimulated p-Akt and pIRS1/2 in MGO-administered mice. Liver tissues were obtained from mice 10 min after insulin injection, and western blot was performed for p-Akt and pIRS1/2. (a) A representative blot is shown. The intensity of band was quantified using the Image Lab software. (b) Quantification of p-Akt/actin from (a). (c) Quantification of p-IRS/actin from (a). Data are shown as mean ± SD (*n* = 3, independent experiments); ^∗∗∗^*p* < 0.005. CON: control; MGO: methylglyoxal; MGO+PCS 200: methylglyoxal+PCS extract 200 mg/kg; MGO+PCS 500: methylglyoxal+PCS extract 500 mg/kg; MGO+AG: methylglyoxal+aminoguanidine.

To further investigate whether the PCS extract could ameliorate MGO-induced insulin resistance *in vivo*, we examined the effects of the PCS extract on the insulin signaling pathway in the liver tissue by western blotting. The administration of MGO markedly decreased the phosphorylation of Akt and IRS1/2 in liver tissue with insulin stimulation, compared to the CON group. Treatment with the PCS extract (200 and 500 mg/kg) significantly restored p-Akt and p-IRS1/2 expression in the MGO-administered mice (Figures 2(a)–2(c)). These data indicated that PCS exerts protective effects against MGO-induced insulin resistance.

### 3.3. PCS Extract Increases Insulin-Stimulated Glucose Uptake and the Expressions of p-Akt and p-IRS1/2 in MGO-Treated HepG2 Cells

**Figure 3:**
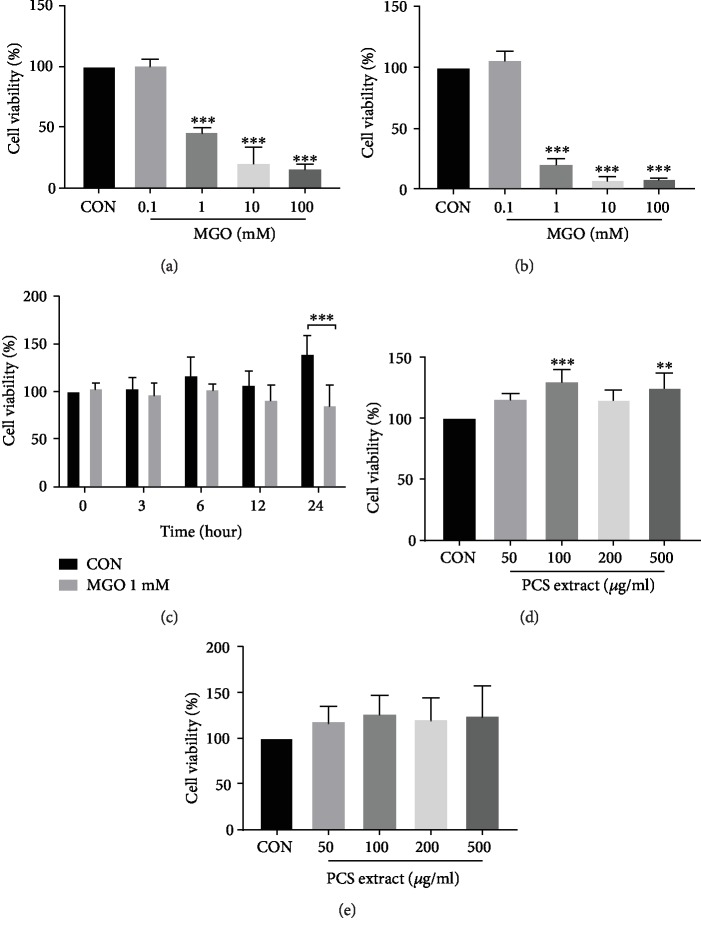
Effects of MGO and PCS extract on cell viability in HepG2 cells. HepG2 cells were treated with the indicated concentrations of MGO (0.1–1 mM) for 24 h (a) or 48 h (b), and the cell viability was measured by CCK assay. (c) HepG2 cells were treated with MGO (1 mM) for the indicated times (0–24 h), and the cell viability was measured by CCK assay. HepG2 cells were treated with the indicated various concentrations of PCS extract (50–500 *μ*g/mL) for 24 h (d) or 48 h (e). The cell viability was measured by CCK assay. Data are shown as mean ± SD (*n* = 3). ^∗∗^*p* < 0.01, ^∗∗∗^*p* < 0.005 versus CON group. CON: control; MGO: methylglyoxal.

To determine the conditions under which MGO can induce insulin resistance without cytotoxicity in HepG2 cells, the cells were treated with various concentrations of MGO (0.1–100 mM) for 24 h or 48 h, and cell viability was evaluated. The proportion of viable cells was significantly decreased by 1 mM MGO at both times, with a dose-dependent decrease with increasingly greater doses (Figures 3(a) and 3(b)). We then checked the cell viability after treatment with 1 mM MGO for different incubation times (0–24 h). The number of viable cells was significantly decreased at 24 h after the treatment (Figure 3(c)). Therefore, we chose the conditions of 1 mM MGO for 12 h, in which the number of viable cells was not reduced, for the subsequent experiments. In addition, HepG2 cells were treated with various concentrations of the PCS extract (50–500 *μ*g/mL) for 24 h or 48 h to check whether the PCS extract displayed any cytotoxicity in HepG2 cells before *in vitro* experiments. PCS extracts did not show any cytotoxicity at all tested concentrations and time points (Figures 3(d) and 3(e)).

**Figure 4:**
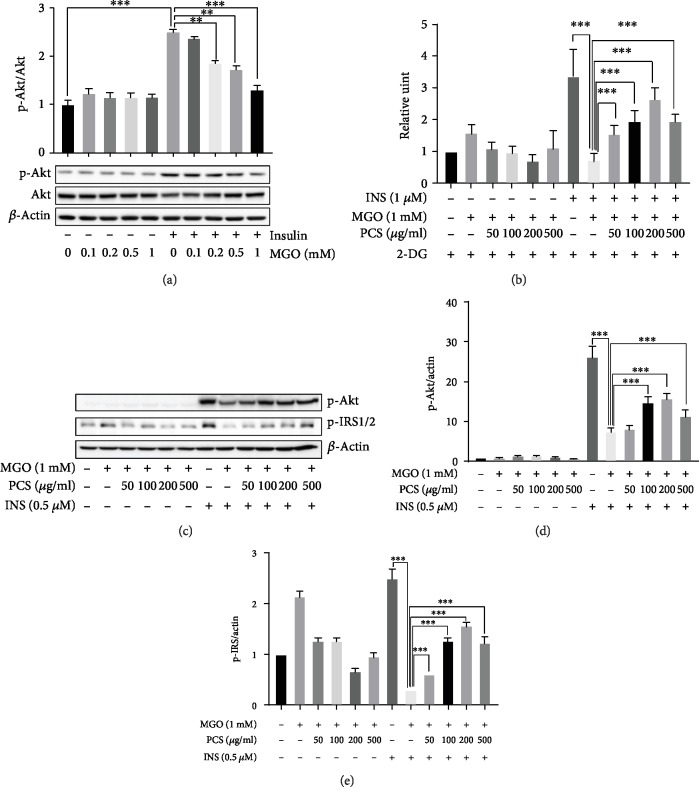
PCS extract recovers the decreased insulin-stimulated glucose uptake and expressions of p-Akt and pIRS1/2 in HepG2 cells. (a) HepG2 cells were treated with MGO (0.1–1 mM, 12 h of treatment) and then stimulated with 0.5 *μ*M insulin for 30 min. The cells were harvested, and the expression of p-Akt was examined by western blotting. (b) Glucose uptake was measured using glucose uptake colorimetric assay kit. (c) The expression of p-Akt and pIRS1/2 was examined by western blotting. Cells were stimulated with 0.5 *μ*M insulin for 30 min. A representative blot is shown. (d, e) The intensity of band was quantified using Image Lab software. (d) Quantification of p-Akt/actin from Figure 2(c). (e) Quantification of p-IRS/actin from Figure 2(c). Data are shown as mean ± SD (*n* = 3, independent experiments); ^∗∗∗^*p* < 0.005. The figure is representative of three independent experiments. CON: control; MGO: methylglyoxal; INS: insulin.

We examined whether MGO induces insulin resistance in HepG2 cells. HepG2 cells were treated with various concentrations of MGO (0.1, 0.2, and 0.5, 1 mM) for 12 h, and p-Akt expression was examined by western blotting. The treatment with 1 mM MGO significantly decreased the insulin-stimulated phosphorylation of Akt compared to the cells without MGO treatment (Figure 4(a)). To investigate whether the PCS extract produced any beneficial effects on MGO-induced insulin resistance in HepG2 cells, we first examined the effects of the PCS extract on glucose uptake in HepG2 cells. Treatment with MGO significantly decreased the insulin-stimulated glucose uptake compared to the cells receiving no MGO treatment. The PCS extract significantly recovered the decreased glucose uptake in the MGO-treated cells from 50 to 200 *μ*g/mL, in a dose-dependent manner, but less effectively at 500 *μ*g/mL (Figure 4(b)). Next, the effects of the PCS extract on the insulin signaling pathway were determined by western blotting. MGO treatment dramatically suppressed the insulin-stimulated expression of p-Akt and p-IRS1/2, compared to the cells receiving no MGO treatment. The treatment with PCS extracts significantly reversed this suppression in the MGO-treated cells, similar to the results observed in the glucose uptake experiment (Figures 4(c)–4(e)). Collectively, these results indicated that the PCS extract attenuated MGO-induced insulin resistance in HepG2 cells.

### 3.4. PCS Extract Inhibits MGO-Induced AGE Formation and Displays an Antiglycan Effect

**Figure 5:**
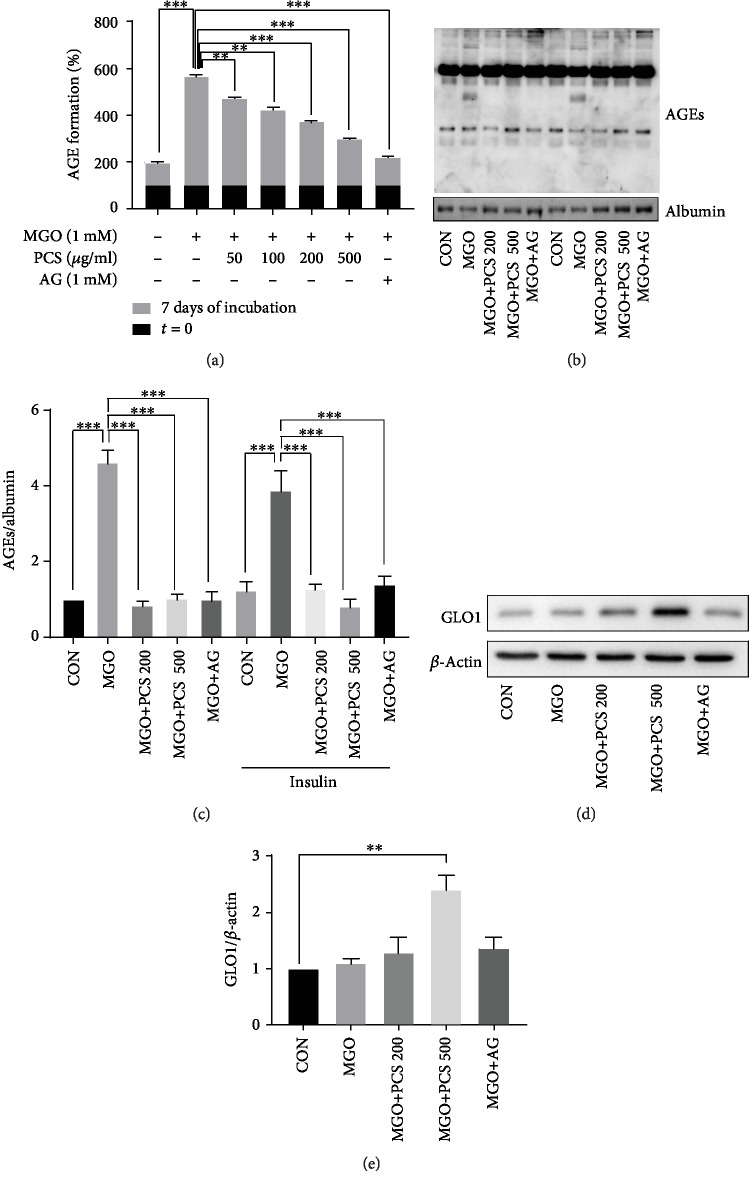
Antiglycan effect of PCS extract on MGO-induced AGE formation. (a) BSA (5 mg/mL) was mixed with 1 mM of MGO in PBS (pH 7.4) in the presence or absence of PCS extracts with concentrations of 50, 100, 200, and 500 *μ*g/mL or 1 mM AG and incubated at 37°C for 7 days. AGE levels were determined by measuring fluorescence at the excitation/emission wavelengths of 355/460 nm. Data are shown as mean ± SD (*n* = 3). (b) Serum was obtained and AGE levels were analyzed by western blotting. The representative blot is shown from three independent experiments. CON: control; MGO: methylglyoxal; MGO+PCS 200: methylglyoxal+PCS extract 200 mg/kg; MGO+PCS 500: methylglyoxal+PCS extract 500 mg/kg; MGO+AG: methylglyoxal+aminoguanidine. (c) Quantification data of (b). (d) Representative image of western blot of GLO1 in liver tissue. (e) Quantification data of (d); ^∗∗^*p* < 0.01, ^∗∗∗^*p* < 0.005.

The formation of AGE has been reported to contribute to inflammation and oxidative stress, which are associated with insulin resistance [6, 9, 22]. We examined whether the PCS extract has any inhibitory effects on AGE formation. Incubation of MGO with BSA for 7 days markedly increased the AGE levels, compared to the group with no added MGO. The PCS extracts (50–500 *μ*g/mL) showed inhibitory effects on AGE formation in a dose-dependent manner. The level of AGEs was also significantly decreased by the addition of AG, a positive control and an inhibitor of AGE formation, compared to the group only treated with MGO (Figure 5(a)).

The levels of AGEs in the sera of the PCS extract-treated mice were then determined by western blotting. The expression of AGEs was indicated as the glycated proteins in the membrane. AGE expression was markedly enhanced in the MGO-administered group as compared to the CON group. As expected, administration of the PCS extract or AG significantly suppressed the expression of AGEs in the MGO-administered mice (Figures 5(b) and 5(c)). These findings suggested that the PCS extract inhibited MGO-induced AGE formation *in vitro* and *in vivo*.

To investigate whether PCS extract has an effect on the expression of GLO1, an enzyme known to play a major role in MGO degradation, GLO1 expression was checked in the liver tissue. PCS extract significantly increased the expression of GLO1 protein at a dose of 500 mg/kg in MGO-administered mice (Figures 5(d) and 5(e)).

### 3.5. PCS Extract Reduces ROS Production and Increases the Expression of Antioxidant Enzymes in MGO-Treated HepG2 Cells

**Figure 6:**
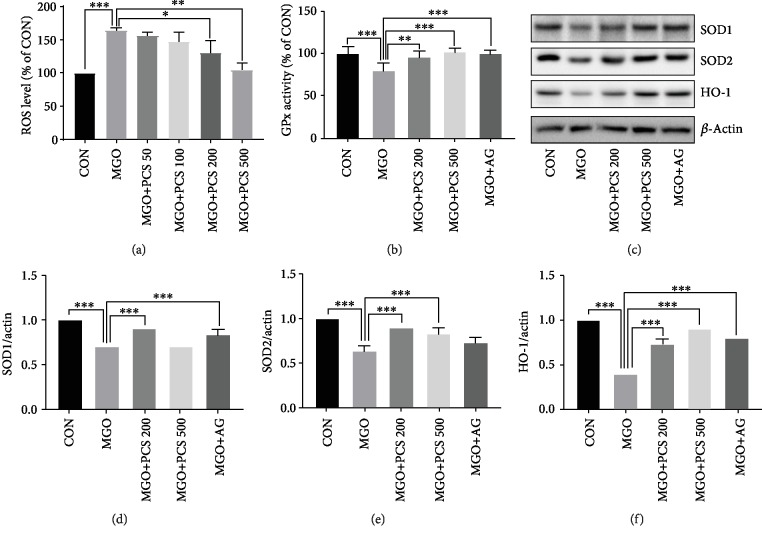
Effects of PCS extract on MGO-induced ROS production in HepG2 cells and the expression of antioxidant enzyme in liver tissue of MGO-administered mice. (a) HepG2 cells were pretreated with the indicated concentration of PCS extract (50–500 *μ*g/mL), followed by treatment with MGO (1 mM) for 1 h. Fluorescent intensity was measured after staining with CM-H_2_DCFDA (10 *μ*M, 30 min). Data are shown as mean ± SD (*n* = 3). (b) GPx activity in liver tissue was measured by GPx assay kit. Data are shown as mean ± SD (*n* = 8). (c) Protein expressions of SOD1, SOD2, and HO-1 were examined by western blotting. A representative blot is shown. (d–f) The intensity of bands was quantified using Image Lab software. Quantification of SOD1/actin (d), SOD2/actin (e), and HO-1/actin (f) from (c). Data are shown as mean ± SD. ^∗^*p* < 0.05, ^∗∗^*p* < 0.01, and ^∗∗∗^*p* < 0.005. CON: control; MGO: methylglyoxal; MGO+PCS 200: methylglyoxal+PCS extract 200 mg/kg; MGO+PCS 500: methylglyoxal+PCS extract 500 mg/kg.

High levels of ROS can cause insulin resistance [23] and AGE can induce ROS production. We examined whether the PCS extract reduced ROS generation in MGO-treated HepG2 cells. We first examined effects of MGO on ROS production in HepG2 cells. Treatment with 1 mM MGO induced ROS production, and pretreatment with different concentrations of the PCS extract (50–500 *μ*g/mL) for 24 h reduced ROS production in a dose-dependent manner (Figure 6(a)).

We then examined the effects of the PCS extract on the activity of the antioxidant enzyme GPx in liver tissues of mice. Administration of MGO caused a significant decrease in GPx activity compared to the CON group. However, treatment with the PCS extract or AG recovered the activity of GPx in MGO-administered mice (Figure 6(b)). In addition, when we examined the expression of the antioxidant enzymes superoxide dismutase (SOD1, SOD2) and heme oxygenase-1 (HO-1) in the liver tissue by western blotting, the expression of these proteins was significantly decreased in MGO-administered mice but was significantly recovered by the PCS extract treatment (Figures 6(c)–6(f)). Collectively, these findings suggested that the PCS extract reduced ROS production and increased the expression of the antioxidant enzymes in MGO-administered mice.

### 3.6. PCS Extract Decreases Expressions of Inflammatory Cytokines and Inflammatory Signaling Molecules in MGO-Administered Mice

**Figure 7:**
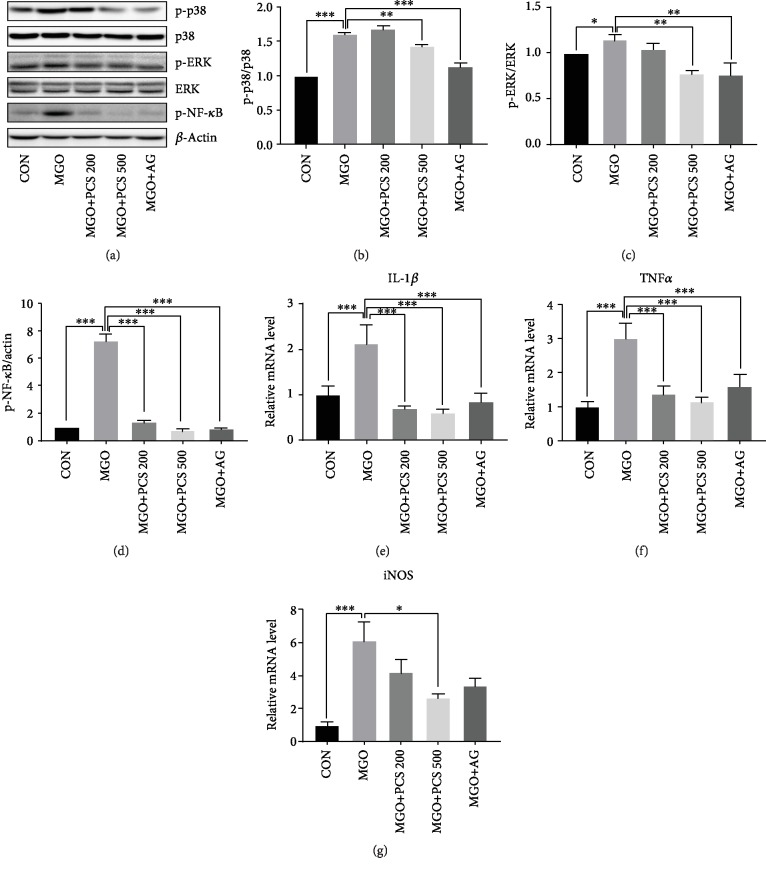
Effects of PCS extract on the expression of inflammatory signaling molecules and mRNA levels of IL-1*β*, TNF-*α*, and iNOS in MGO-administered mice. (a) Liver tissues were obtained, and the expression of pNF-*κ*B p65, p-ERK, and p-p38 was examined by western blotting. A representative blot is shown from three independent experiments. (b–d) The intensity of bands was quantified using Image Lab software. Quantification of p-p38/p38 (b), p-ERK/ERK (c), and p-NF-*κ*B/actin (d) from (a). Data are shown as mean ± SD. mRNA expression of IL-1*β* (e), TNF-*α* (f), and iNOS (g) was examined by qRT-PCR analysis. Data are shown as mean ± SD (*n* = 8). ^∗^*p* < 0.05, ^∗∗^*p* < 0.01, and ^∗∗∗^*p* < 0.005. CON: control; MGO: methylglyoxal; MGO+PCS 200: methylglyoxal+PCS extract 200 mg/kg; MGO+PCS 500: methylglyoxal+PCS extract 500 mg/kg; MGO+AG: methylglyoxal+aminoguanidine.

Binding of AGEs to RAGE activates NF-*κ*B, contributing to the expression of inflammatory cytokines and ROS production [24]. We examined the effects of the PCS extract on the activation of NF-*κ*B and upstream signaling pathways including ERK and p38 in liver tissue by western blotting. MGO administration increased the expression of p-NF-*κ*B p65, p-ERK, and p-p38 compared to the CON group. Treatment with 500 mg/kg PCS extract significantly suppressed all these deleterious effects on the inflammatory signaling induced by MGO, whereas treatment with 200 mg/kg and 500 mg/kg PCS extract markedly decreased NF-*κ*B activation. AG also exhibited inhibitory effects on the expression of inflammatory signaling molecules in the MGO-administered mice (Figures 7(a)–7(d)).

The activation of NF-*κ*B stimulates the expression of proinflammatory cytokines, such as tumor necrosis factor-alpha (TNF-*α*) and interleukin- (IL-) 1*β*. Therefore, we examined the effects of the PCS extract on the mRNA expression of proinflammatory cytokines in the liver tissue of MGO-administered mice. The mRNA levels of IL-1*β*, TNF-*α*, and induced nitric oxide synthase (iNOS) were significantly increased in the liver of the MGO-administered mice, and this increase was significantly inhibited by the PCS extract in a dose-dependent manner (Figures 7(e)–7(g)).

## 4. Discussion

MGO is the most important and well-studied precursor of AGEs [25]. This highly reactive dicarbonyl metabolite reacts with arginine or lysine residues of proteins to form AGEs [20, 25]. Accumulation of AGEs has been reported to contribute to inflammation and oxidative stress via the overproduction of ROS, which are recognized as contributing factors in insulin resistance [22]. This study is aimed at investigating the ameliorative effects of the PCS extract on the insulin resistance induced by MGO.

Insulin resistance is generally defined as a reduced response of target tissues, such as the skeletal muscle, liver, and adipocytes, to insulin [3]. Insulin exerts its effects by binding to the insulin receptors, which induce the phosphorylation of the tyrosine residues on insulin receptor substrate (IRS) proteins. The phosphorylation of IRS results in the activation of Akt, which stimulates the translocation of glucose transporter (GLUT) from the cytosol to the cell membrane, facilitating glucose uptake into cells [26, 27]. Previous studies indicated that treatment with 0.5–2.5 mM MGO resulted in insulin resistance in L6 cells and HepG2 cells [28, 29]. In addition, several studies have reported the direct effects of AGEs on insulin action *in vitro* and *in vivo* [9, 30, 31]. In the present study, PCS extract significantly reduced MGO-induced insulin resistance, resulting in the recovery of glucose uptake in the insulin-stimulated HepG2 cells. In addition, we also investigated the effect of PCS extract on MGO-induced insulin resistance *in vivo*. To induce insulin resistance, we used a supraphysiological dose of MGO for oral administration, which is a limitation to our study. Nevertheless, the PCS extract significantly improved glucose tolerance and insulin sensitivity in MGO-administered mice. Consistently, phosphorylation of biomolecules in the insulin signaling pathway, such as Akt and IRS1/2, was increased in the liver tissue of MGO-administered mice treated with PCS extract. Collectively, these findings indicate that the PCS extract ameliorated the MGO-induced insulin resistance *in vitro* and *in vivo*.

MGO can form AGEs, and AGEs might be the messenger in the signaling that causes insulin resistance. Blockage of AGE formation can be considered as a promising therapeutic strategy to counteract the glycation-induced insulin resistance. Therefore, we first investigated whether the PCS extract can affect AGE formation induced by MGO. We evaluated the inhibitory effects of the PCS extract on AGE formation induced by MGO. MGO induced a significant increase in the formation of AGEs after 7 days of incubation with BSA. The PCS extract (50–500 *μ*g/mL) inhibited the MGO-induced AGE formation in a dose-dependent manner. *In vivo*, treatment with PCS extract significantly suppressed the serum AGE level in MGO-administered mice. These findings suggest that PCS extract can inhibit AGE formation.

Many natural products, including the extracts of herbal plants, mung beans, peanuts, or natural compounds, such as quercetin and myricitrin, have been reported to inhibit the formation of AGEs induced by MGO [21, 32–35]. Moreover, it has been reported that natural products block the MGO-induced AGE formation through the trapping of MGO [21, 32]. This MGO trapping mechanism by flavonoids from natural products involves the trapping of the reactive dicarbonyl MGO species at the two unsubstituted positions 6 and 8 in the A-ring, the major active sites of flavonoids. This results in the formation of mono- and di-MGO adducts, especially under alkaline conditions, owing to the high electron density upon dissociation of a hydrogen atom from two hydroxyl groups [36]. It has also been reported that natural extracts, such as *Arachis hypogaea* (peanut) extract and *Garcinia indica* fruit rind extract, inhibit AGE formation as effective chain-cleavage antioxidants or as free radical scavengers by chelating trace metal ions included in the phosphate buffer, which catalyze glycation [21, 37]. We believe that inhibition of AGE formation by the PCS extract may have resulted via a similar mechanism.

In the physiological environment, MGO is mainly formed as a byproduct of glycolysis and is detoxified to D-lactate by the glyoxalase system. In this process, glyoxalase I (GLO1) is the most crucial enzyme [38]. In this study, PCS extract treatment increased GLO1 protein levels in MGO-treated cells. This indicates that PCS extract may inhibit glycation effect by inhibiting AGE formation through the trapping of MGO and also due to the degradation of MGO by GLO1.

The AGE-RAGE interaction stimulates various signaling cascades, including MAPK, such as p38 and ERK-1/2, and this signaling cascade can activate NF-*κ*B, which, in turn, is followed by an increased expression of cytokines [38]. It has been reported that AGEs stimulate the production of proinflammatory cytokines, such as TNF-*α* and IL-1*β*, via activation of the NF-*κ*B signaling pathway [39]. Our results showed that MGO treatment significantly increased the phosphorylation of NF-*κ*B, ERK, and p38, as well as the mRNA expression of TNF-*α*, IL-1*β*, and iNOS. The treatment with PCS extract significantly suppressed all these deleterious effects induced by MGO in the liver tissue of MGO-administrated mice. Therefore, PCS extract inhibited AGE formation, thus inhibiting the AGE-RAGE signaling pathway, and contributed to the inhibition of inflammatory molecule expression.

TNF-*α* and IL-1*β* directly induce ROS production and also increase the expression of iNOS, which can produce ROS and RNS [40, 41]. Therefore, we checked the effects of the PCS extract on MGO-induced ROS production in HepG2 cells. As expected, treatment with PCS extract reduced the ROS production in MGO-treated HepG2 cells in a dose-dependent manner. Additionally, PCS extract treatment recovered the activity of GPx and increased protein expression of antioxidant enzymes including SOD1, SOD2, and HO-1 in the liver tissue of the MGO-administered mice. These findings are consistent with the suggestion that PCS extract also reduces the ROS level by the downregulation of the controlling antioxidant enzymes, as the removal of ROS is regulated by several antioxidant enzymes, including GPx, SOD1, SOD2, and HO-1 [23, 42]. Moreover, consistent with these results, previous studies have reported that PCS extract possesses antioxidant effects and protects against mitochondrial dysfunction induced by oxidative stress in HepG2 cells [15].

In conclusion, PCS extract attenuated the MGO-induced insulin resistance in HepG2 cells and also in an animal model. PCS extract suppressed AGE production by MGO trapping and MGO degradation and subsequently inhibited AGE-RAGE signaling pathways, contributing to the attenuation of insulin resistance. These findings implicate PCS extract as a potential candidate for the prevention and treatment of insulin resistance.

## Figures and Tables

**Table 1:** List of primers used for qRT-PCR.

Gene	Forward/reverse primers (5′-3′)
Cyclophilin B	TGCCATCGCCAAGGAGTAG
	TGCACAGACGGTCACTCAAA
SOD1	GTGATTGGGATTGCGCAGTA
	TGGTTTGAGGGTAGCAGATGAGT
SOD2	TTAACGCGCAGATCATGCA
	GGTGGCGTTGAGATTGTTCA
HO-1	GCCTGCTAGCCTGGTGCAAG
	AGCGGTGTCTGGGATGAGCTA
IL-1*β*	CTACAGGCTCCGAGATGAACAAC
	TCCATTGAGGTGGAGAGCTTTC
TNF-*α*	CCAACGGCATGGATCTCAAAGACA
	AGATAGCAAATCGGCTGACGGTGT
iNOS	GGCAGCCTGTGAGACCTTTG
	TGCATTGGAAGTGAAGCGTTT

## Data Availability

The data and figures used to support the findings of this study are included within the article.
